# Mean fitness is maximized in small populations under stabilizing selection on highly polygenic traits

**DOI:** 10.1101/2025.11.17.688329

**Published:** 2025-11-19

**Authors:** Aaron P. Ragsdale

**Affiliations:** Department of Integrative Biology, University of Wisconsin–Madison

**Keywords:** Stabilizing selection, mean fitness, genetic load, polygenic trait

## Abstract

Stabilizing selection commonly acts on complex traits that affect individual fitness. Here, we relate mean fitness under stabilizing selection to population size and trait architecture, using a simple application of theoretical predictions for the distribution of phenotypic values in a single-trait Gaussian stabilizing selection model. We show that mean fitness is maximized by a finite (often small) population size when the total mutation rate U across trait-affecting loci is reasonably large. Namely, this occurs when $U>V_{M}/8\left(V_{S}+V_{E}\right)$, where $V_{M}$ is the variance of the distribution of effect sizes of new mutations, $V_{S}$ determines the strength of stabilizing selection, and $V_{E}$ is the environmental variance. We validate these predictions using individual-based simulations and briefly discuss their implications for interpreting genetic load and adaptability in small populations.

## Introduction

It is generally understood in conservation and population genetics that a reduced population size poses evolutionary challenges for species survival and individual fitness. In a small population, deleterious variation is allowed to segregate at higher frequencies (Kimura et al., 1963), contributing to genetic load (Haldane, 1937; Crow, 1958), and mildly and moderately deleterious mutations fix more readily, leading to reduced absolute fitness among individuals (Lande, 1994; Lynch et al., 1995a,b). A smaller population size also results in lower levels of standing variation, limiting the response to selection when faced with a novel selective pressure or a changing environment (Lande and Shannon, 1996; Hayward and Sella, 2022).

It has also been established that the mode of selection on individual loci is an important factor in predicted genetic load and standing functional variation. For example, Kimura et al. (1963) showed that mildly deleterious mutations can contribute more to load than strongly deleterious ones, and in some scenarios partially recessive deleterious variation can result in small, finite populations having smaller load than large populations (see also Keller and Waller, 2002). Smaller populations may, in certain scenarios, more effectively purge recessive deleterious variants, as those alleles are more frequently exposed to selection as homozygotes (Kyriazis et al., 2021). Nonetheless, many examples from natural systems, domesticated species, and lab experiments show that reduced long-term $N_{e}$ corresponds to increased measures of genetic load and reduced overall diversity (e.g., Paland and Schmid, 2003; Willi et al., 2013; Pekkala et al., 2014, and recently reviewed in Robinson et al. (2023)).

In much of this literature, the phenotype of interest is fitness itself. To make model predictions, the selective effects of the many deleterious mutations that reduce fitness are typically combined multiplicatively, and selection acts directionally on individual fitness. However, for many traits that contribute to fitness variation in natural populations, stabilizing selection acts to maintain phenotypic values near some intermediate optimum (Sanjak et al., 2018; Sella and Barton, 2019; Koch et al., 2024; Patel et al., 2025). Instead of directional negative selection on unconditionally deleterious variants, selection at trait-affecting loci resembles underdominance (Robertson, 1956) – stabilizing selection acts to reduce the genetic variance of a trait, which manifests as selection against the minor allele.

With stabilizing selection on a quantitative trait, how do population size and trait architecture interact to affect mean fitness? Does our intuition still hold, in which small populations have decreased mean fitness? We would expect that stabilizing selection more effectively maintains a population’s mean phenotype near the optimal trait value when the population is large. At the same time, the genetic variance of a trait increases with the population size, and this depends also on the rate and effects of new mutations and the strength of stabilizing selection. Below, we combine these predictions to show that mean fitness is maximized in small populations when the total rate of mutations U affecting the trait is sufficiently large, a condition that is likely met for many complex traits of interest.

## Mean fitness with stabilizing selection on one trait

We model individual fitness following a standard Gaussian stabilizing selection model, with inverse strength of stabilizing selection $V_{S}$, so that an individual with the optimal phenotype has fitness one. The fitness of an individual with trait value p, relative to the optimal phenotype, is then

(1) $$w\left(p\right)=\exp\left(\frac{-{\left(p\;-\;opt\right)}^{2}}{2V_{S}}\right).$$

In what follows, we set the optimal phenotype to zero. Given some distribution f(p) of phenotypic values among individuals in the population, the mean fitness of the population is found by taking the integral

(2) $$\overset{‾}{w}=\int_{-\infty }^{\infty }w(p)f(p)\;dp.$$

**Figure A1:**
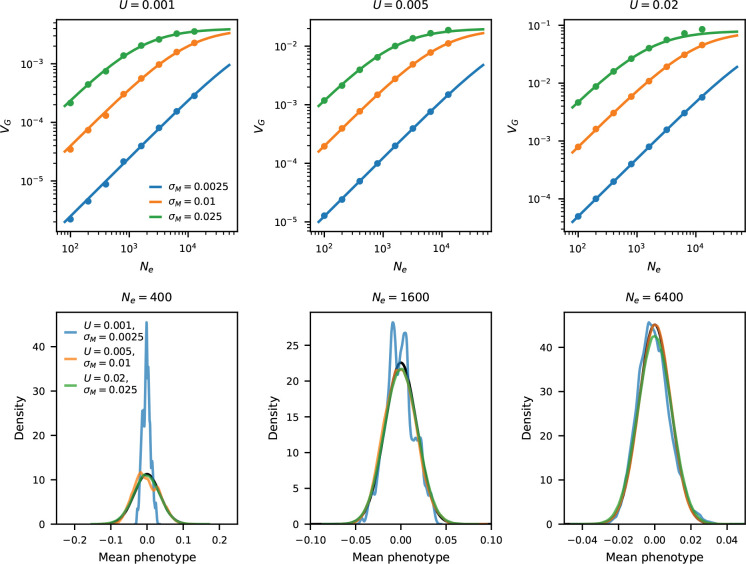
Model predictions are robust across a wide range of parameters. Top row: The SHOC model is a good approximation for $V_{G}$, unless U is large enough to break assumptions of linkage equilibrium. Dots indicate averages over replicates of individual-based forward-in-time simulations. Bottom row: $\overset{‾}{p}∼𝒩\left(0,V_{S}/2N_{e}\right)$ is good approximation, with the normal distribution density shown as a black curve. This breaks down for very small population sizes (with $N_{e}$ in the hundreds) and lower mutation rates.

We assume that the distribution of phenotypes is close to (but not necessarily at) the optimum. With many mutations contributing to the trait, phenotypes will be approximately normally distributed with variance $V_{P}$ and mean $\overset{‾}{p}$: that is, $f(p,\overset{‾}{p})=𝒩\left(\overset{‾}{p},V_{P}\right)$. Furthermore, $\overset{‾}{p}$ is expected to fluctuate around the optimum, and the displacement from the optimum (δ) is normally distributed with mean zero and variance $\frac{V_{S}}{2N_{e}}$ (Simons et al., 2018, and see Fig. A1): that is, $\delta (\overset{‾}{p})=𝒩\left(0,V_{S}/2N_{e}\right)$.

Then the expected mean fitness of the population, integrating over the distribution of $\overset{‾}{p}$, is

(3) $$\begin{array}{l} E[\overset{‾}{w}]=\int_{-\infty }^{\infty }\delta (\overset{‾}{p})\int_{-\infty }^{\infty }\;w(p)f(p,\overset{‾}{p})\;dp\;d\overset{‾}{p}\\ \;={\left(\frac{2N_{e}V_{S}}{V_{S}\;+\;2N_{e}\left(V_{P}\;+\;V_{S}\right)}\right)}^{1/2}. \end{array}$$

In the limit $N_{e}\to \infty$, we recover

(4) $$E[\overset{‾}{w}]\to {\left(\frac{V_{S}}{V_{P}\;+\;V_{S}}\right)}^{1/2},$$

as deviations of the population mean phenotype approach zero with increasing population size.

## The stochastic house of cards model

We ignore environmental effects, noting that the environmental variance can be absorbed into the fitness function (Appendix A, and see Simons et al., 2018). We further assume linkage equilibrium between loci, and we ignore epistasis, dominance and pleiotropy. We discuss the relaxation of some of these assumptions below.

Under these assumptions, the phenotypic variance is equal to the additive genetic variance, $V_{G}$. Generally, $V_{G}$ will depend on the total mutation rate U(=μL, with μ the per-base haploid rate, L the target size for the trait), the distribution of mutation effect sizes (assumed to be $𝒩\left(0,V_{M}\right)$), and the strength of selection (determined by $V_{S}$). If effect sizes are large,

(5) $$V_{G}\approx 4UV_{S},$$

a classic result originally due to Latter (1960). In the opposite limit, when effect sizes are weak, $V_{G}$ is linear in the population size-scaled mutation rate and mutational variance (Lande, 1976), so

(6) $$V_{G}\approx 4N_{e}UV_{M}.$$

The stochastic house of cards (SHOC) model interpolates these two regimes (see Ch. 22 in Walsh and Lynch, 2018; Bürger et al., 1989), so that

(7) $$V_{G}\approx \frac{4UV_{S}}{1\;+\;\frac{V_{S}}{N_{e}V_{M}}}.$$

This has been shown to be good approximation at steady state across a wide range of mutational effect sizes.

Replacing this expression for $V_{P}$ in the prediction for $E[\overset{‾}{w}]$ (Eq. 3), we obtain

(8) $$E[\overset{‾}{w}]={\left(\frac{2N_{e}}{1\;+\;2N_{e}\left(1\;+\;\frac{4N_{e}UV_{M}}{N_{e}V_{M}\;+\;V_{S}}\right)}\right)}^{1/2}$$

(9) $$\approx 1-\frac{2N_{e}V_{M}U}{N_{e}V_{M}\;+\;V_{S}}-\frac{1}{4N_{e}}.$$

**Figure 1:**
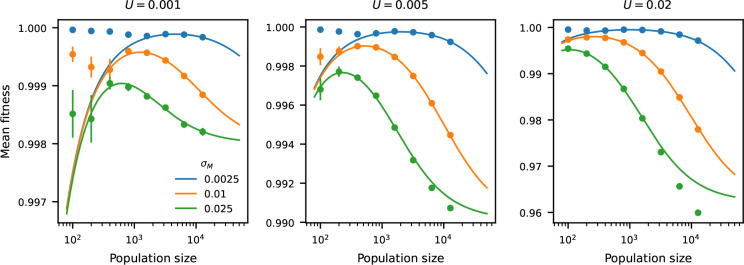
Small population sizes can maximize mean fitness when polygenic traits are under stabilizing selection. Predictions for mean fitness within populations (Eq. 8, solid curves) show that mean fitness can decline as population sizes increase. Predictions are validated using individual-based simulations (points with error bars indicating 95% confidence intervals over replicates), varying population size, total mutation rate, and the variance ($\sigma_{M}^{2}$) of effect sizes for new mutations (Thornton, 2019). $V_{S}$ is set to 1 in all simulations. When population sizes are very small 𝒪(100)), simulatinos deviate from predictions because the predictions for the fluctuations of mean phenotype around the optimum ($𝒩\left(0,V_{S}/2N_{e}\right)$) break down (Fig. A1D–F). For strong mutational effects and higher mutation rates, predictions break down because the SHOC model underestimates $V_{G}$ (Fig. A1A–C).

This prediction (Eq. 8) is accurate across a wide range of model parameters (Fig. 1). Thus, as $N_{e}$ becomes large, the genetic load is approximately 2U (though this is an overestimate), and as $N_{e}$ becomes small, the genetic load is approximately $1/4N_{e}$.

## Condition for increased mean fitness with finite population size

Taking the derivative of $E[\overset{‾}{w}]$ (Eq. 8) with respect to $N_{e}$ is simple (if a bit tedious), and the numerator is quadratic in $N_{e}$. We can set it equal to zero and quickly solve for the effective size that maximizes expected mean fitness. This emits a positive solution,

(10) $$N_{e}=\frac{V_{S}\left(1\;+\;\sqrt{\frac{8UV_{S}}{V_{M}}}\right)}{8UV_{S}\;-\;V_{M}},$$

when $8UV_{S}-V_{M}>0$, that is, if the total mutation rate at trait-affecting loci

(11) $$U>\frac{V_{M}}{8V_{S}},$$

or if the target size is large enough,

(12) $$L>\frac{V_{M}}{8\mu V_{S}}.$$

In the presence of uncorrelated environmental noise contributing to phenotypes, we can replace $V_{S}$ with $V_{S}+V_{E}$ (see Appendix A), so a finite $N_{e}$ maximizes mean fitness in the population when

(13) $$U>\frac{V_{M}}{8\left(V_{S}\;+\;V_{E}\right)}.$$

## Conclusions

Under equivalent trait architecture and strength of stabilizing selection, smaller populations harbor lower genetic variance (Eq. 7), but the average phenotype tends to drift farther from the optimum ($Var(\overset{‾}{p})=V_{S}/2N_{e}$). If U is sufficiently large, then mutation-stabilizing selection balance results in the genetic variance of the trait to be high enough in large populations so that the average fitness across all individuals is lower relative to a small population.

Are the conditions in which this occurs (Eq. 11) relevant to complex traits in natural populations? In a recent analysis of human GWAS results from 95 traits, Simons et al. (2025) find that L and $h^{2}$ both vary considerably across traits, but they estimate L is typically of order 10^6^ or larger. Distributions of effect sizes span multiple orders of magnitude, although $V_{M}$ is difficult to estimate for any given trait. We may instead consider the relationship

$$8U\frac{V_{S}}{V_{P}}>\frac{V_{M}}{V_{P}},$$

and whether we expect it to be satisfied. For human quantitative traits, $V_{S}/V_{P}$ has been estimated to be ~ 60 (28–173), implying weak but non-negligible stabilizing selection (Sanjak et al., 2018). If we assume a per-base mutation rate of $\mu ∼10^{-8}$ and $L≳10^{6}$ (and ignoring environmental variance), the quantity on the left should easily exceed 1. And while $V_{M}$ may be unknown, we should expect $V_{M}/V_{P}$ to fall below 1, implying that our condition is likely met.

Such large mutational target sizes for complex traits in humans (with $L∼𝒪\left(10^{6}\right)$ or greater) implies extensive pleiotropy across trait architectures. The model considered here is simple – a single trait under direct stabilizing selection. Understanding fitness variation within populations with highly polygenic, pleiotropic trait architectures will continue to be an important direction for theoretical and empirical work. Direct applications to human trait architectures may be challenging, because neither multivariate trait fitness functions nor the distribution of mutational effects across traits are expected to be isotropic, and the their covariances are largely unknown. Here, we have assumed a Gaussian distribution of effects for a single trait, which has been commonly deployed in the literature. The genetic load may additionally be sensitive to the precise distribution of effects (De Vladar and Barton, 2014), complicating the generality of these results.

Demographic and selection processes are not expected to remain at steady state. In particular, phenotypic optima can change, and a population’s rate of approach to the new optimum is determined by the genetic variance of the trait (Lande, 1994; Hayward and Sella, 2022). A population with reduced genetic variance, as is the case in small populations, may be challenged to readily adapt in a rapidly changing environment. As with pleiotropic selection, understanding fitness and trait dynamics in a continually changing environment and for non-panmictic populations is a relevant direction for future work (Bertram and Shafiei, 2025; Veller and Muralidhar, 2025).

## Data Availability

Python scripts and a Mathematica notebook to recreate these results are availabile at https://github.com/apragsdale/stabilizing_selection_Ne.

## Appendix

### A Accounting for environmental variance

We model phenotypes as P=G+E, where $E∼𝒩\left(0,V_{E}\right)$ and is independent of G. Then $V_{P}=V_{G}+V_{E}$, and we argue that $V_{E}$ can be absorbed into $V_{S}$ in the fitness function be integrating over environmental backgrounds (Lande, 1975, and see Eq. A1 from Simons et al. (2018)):

(A1)
$$w_{e}(g)=\frac{1}{{\left(1\;+\;V_{E}/V_{S}\right)}^{1/2}}exp\left(\frac{-(g\;-\;opt{)}^{2}}{2\left(V_{S}\;+\;V_{E}\right)}\right).$$

Setting ${\tilde{V}}_{S}=V_{S}+V_{E}$, this can be written as

(A2)
$$w_{e}\left(g\right)={\left(\frac{V_{S}}{{\tilde{V}}_{S}}\right)}^{\frac{1}{2}}\exp\left(\frac{-(g\;-\;opt{)}^{2}}{2{\tilde{V}}_{S}}\right).$$

Now, the distribution of mean genetic values $\left(\overset{‾}{g}\right)$ follows $\delta (\overset{‾}{g})=𝒩\left(0,{\tilde{V}}_{S}/2N_{e}\right)$, and the distribution of genetic values follows $f(g|\overset{‾}{g})=𝒩\left(\overset{‾}{g},V_{G}\right)$. Then integrating over genetic values and the distribution of mean values (as in Eq. 3), we get

(A3)
$$\begin{array}{ll} E[\overset{‾}{w}] & =\int_{-\infty }^{\infty }\;\delta (\overset{‾}{g})\int_{-\infty }^{\infty }\;w_{e}(g)f(g|\overset{‾}{g})\;dpd\;\overset{‾}{p}\\ & ={\left(\frac{V_{S}}{{\tilde{V}}_{S}}\right)}^{1/2}{\left(\frac{2N_{e}{\tilde{V}}_{S}}{{\tilde{V}}_{S}\;+\;2N_{e}(V_{G}\;+\;{\tilde{V}}_{S})}\right)}^{1/2}. \end{array}$$

This takes the same form as Eq. 3 with $V_{S}$ replaced by ${\tilde{V}}_{S}$ and decreased by a factor

$${\left(\frac{V_{S}}{{\tilde{V}}_{S}}\right)}^{1/2}={\left(\frac{V_{S}}{V_{S}\;+\;V_{E}}\right)}^{1/2}.$$

Then, under the SHOC model,

$$V_{G}\approx \frac{4U{\tilde{V}}_{S}}{1\;+\;\frac{{\tilde{V}}_{S}}{N_{e}V_{M}}}.$$

Replacing this expression for $V_{G}$ above and carrying through the steps in the main text, we find that the value of $N_{e}$ that maximizes $E[\overset{‾}{w}]$ is unchanged (Eq. 10). Our condition for a fitness-maximizing finite $N_{e}$ is now

(A4)
$$U>\frac{V_{M}}{8{\tilde{V}}_{S}}=\frac{V_{M}}{8\left(V_{S}\;+\;V_{E}\right)}.$$

### B Simulation methods and validation

Using the forward-in-time simulation software fwdpy11 (Thornton, 2019), we simulated populations of varying size with different mutation and selection parameters, with multiple replicates per parameter set. After allowing the simulation to run for 20N generations to reach quasi-steady state, we stored the distribution of phenotypes and fitnesses across individuals each generation for 4N generations. From this information, we computed mean phenotypes, the variances of phenotypes, and mean fitnesses across all replicates of the same parameter set to compare to model predictions (Fig. 1).

In some parts of parameter space, we noted that simulated values deviated from model predictions, in particular for very small population sizes and separately for high mutation rates and effect sizes in larger populations. The sources of these deviations differed. With high mutation rates (and thus higher polygenicity and a greater density of trait-affecting variable sites along the genome) $V_{G}$ can be larger than predicted under the SHOC model (Fig. A1:B,C with $\sigma_{M}=0.025$). This in turn causes an overestimation of mean fitness. On the other hand, when population sizes and mutation rates are small, the approximation that the mean phenotype of the population is normally distributed around the optimum with variance $\sigma_{M}=0.025$ breaks down (Fig. A1D).
